# Near-death experiences in survivors of cardiac arrest: a study about demographic, medical, pharmacological and psychological context

**DOI:** 10.1186/cc14501

**Published:** 2015-03-16

**Authors:** F Lallier, G Velly, A Leon

**Affiliations:** 1 Centre Hospitalier Universitaire, Reims Cedex, France

## Introduction

Near-death experiences (NDEs) are increasingly being reported as a clear reality of clinical significance. Previous studies, essentially, have been trying to estimate their incidence in various populations, notably after cardiac arrest resuscitation, and to understand the implication of resuscitation characteristics [1,2]. Using the Greyson NDE scale [3], the present retrospective study aimed at exploring cardiac arrest survivors and the correlations between NDE and physiological, medical, psychological and pharmacological context.

## Methods

In a retrospective study from 2005 to 2012, 295 consecutive cardiac patients who were successfully resuscitated after cardiac arrest were enrolled. In total, 204 (69%) were alive during the research period (mean delay: 55 months). A total of 118 (40%), over 18 years, able to answer a short standardized interview were included in the study when they accepted to participate. Demographic, medical, pharmacological and psychological data were recorded and we used the Greyson NDEs scale to identify and characterize NDEs. Descriptive and unifactorial analysis was performed using the Jacknife method and Wald test according to low event frequency.

## Results

From our 118 reports, 20 described a core experience and 18 (15.3%) met the criteria for NDEs (Greyson NDEs total score >6/32 (7 to 19)). Only one patient recounted a negative experience. Regarding the risk factors for NDEs, using univariate analysis, we found for demographic data: woman (CI: 1.11 (1.10 to 1.12), *P *< 0.0001), age under 60 (CI: 1.23 (1.21 to 1.24), *P *< 0.0001), prior knowledge of NDEs (CI: 1.97 (1.95 to 1.99)) and previous NDE (CI: 5.82 (4.19 to 8.08)). According to the history of previous disease, we found an increased risk for pulmonary disease (CI: 1.75 (1.73 to 1.77), rheumatic disease (CI: 3.79 (3.75 to 3.84)), endocrine disease (CI: 1.45 (1.43 to 1.46)), and a decrease for cardiac disease (CI: 0.65 (0.64 to 0.66)), psychiatric disease (CI: 0.71 (0.69 to 0.72)) and digestive tract disease (CI: 0.71 (0.69 to 0.72)). For previous pharmacological treatment we found a decrease of risk for all classes and particularly when two drugs were simultaneously given (CI: 0.37 (0.36 to 0.38)).

## Conclusion

Although our study has a number of methodological limitations, these results about incidence in cardiac arrest survivors corroborate previous retrospective reports. It is possible that every cardiac arrest survivor has had to live a NDE, regardless of brain mechanisms associated with experience, but only some patients remember it. If some chronic medications, such as benzodiazepine, may decrease memorization, the role of the elements of the clinical context about NDE during resuscitation is speculative
